# Misinterpretation of High-Resolution Manometry Leading to Inappropriate Treatment of Achalasia: A Diagnostic Challenge

**DOI:** 10.7759/cureus.89206

**Published:** 2025-08-01

**Authors:** Kyle Schneider, David S Braun, Prashant Kedia

**Affiliations:** 1 Internal Medicine, Methodist Dallas Medical Center, Dallas, USA; 2 Gastroenterology, Methodist Health System, Dallas, USA

**Keywords:** achalasia, endoflip, gastroenterology, high-resolution manometry, poem

## Abstract

Achalasia is a motility disorder of the esophagogastric junction outflow, characterized by impaired lower esophageal sphincter (LES) relaxation and loss of normal peristalsis of the esophageal smooth muscle. The common clinical manifestations of achalasia include dysphagia of both solids and liquids, regurgitation of undigested food and saliva, and chest pain. It shares symptoms with gastroesophageal reflux disease (GERD), such as a retrosternal burning sensation and dysphagia, which can delay the diagnosis. Several modalities are useful in establishing the diagnosis of achalasia, including high-resolution manometry (HRM), barium esophagram (BE), and upper endoscopy.

Despite potential issues with HRM, it remains the gold standard for diagnosing achalasia, underscoring the importance of proper technique and interpretation. Improper probe placement can lead to inaccurate diagnoses. Here, we present the case of a patient with achalasia who was misdiagnosed with GERD and underwent an inappropriate Toupet fundoplication, who eventually required peroral endoscopic myotomy (POEM) as a salvage treatment to relieve their symptoms.

## Introduction

Achalasia is a motility disorder of the esophagogastric junction (EGJ) characterized by impaired lower esophageal sphincter (LES) relaxation and loss of normal esophageal peristalsis. Its pathophysiology involves degeneration of esophageal smooth muscle innervation, with a loss of ganglion cells in the myenteric plexus. The underlying etiology of primary achalasia is unknown. Achalasia, a relatively uncommon condition with a prevalence of about 0.1%, shares multiple clinical symptoms with gastroesophageal reflux disease (GERD), which is much more common, with a prevalence of up to 25% the United States [1]. While achalasia and GERD have distinct pathophysiological mechanisms, they share overlapping clinical features. The cardinal symptoms of achalasia include dysphagia to both solids and liquids and regurgitation of undigested food and saliva, while patients with GERD classically present with pyrosis and regurgitation of gastric contents. However, atypical presentations and diagnostic challenges can lead to misdiagnosis, with potential implications for treatment selection.

Several diagnostic modalities are used to differentiate between achalasia and GERD. The American Society of Gastrointestinal Endoscopy guidelines recommend early esophagogastroduodenoscopy (EGD) in patients with GERD who have alarm symptoms, such as dysphagia or lack of response to acid-suppressive therapy, to rule out alternative diagnoses [1,2]. In achalasia, EGD typically demonstrates a dilated esophagus with retained food, an LES with a puckered appearance, and unusually increased resistance to the passage of the endoscope. Endoscopic findings related to GERD most commonly show normal esophageal mucosa or varying degrees of erosive esophagitis. Timed barium esophagram (TBE) and high-resolution manometry (HRM) are also commonly used to distinguish achalasia from GERD. TBE findings suggestive of achalasia include aperistalsis, delayed barium emptying, esophageal dilation, and a narrowed EGJ with a “bird-beak” appearance. TBE findings in GERD may also show esophageal dysmotility, but usually without a bird’s beak EGJ, and may be accompanied by over-reflux of contrast and concurrent hiatal hernias. While EGD and TBE are useful adjunctive tests that may augment clinical suspicion for achalasia when classical features are present, they lack the sensitivity and specificity to make a definitive diagnosis, especially in early disease. Thus, the gold standard for diagnosing achalasia is HRM, which identifies increased median relaxation pressure and abnormal peristalsis [3]. Despite its high negative predictive value, HRM can yield false negatives due to various reasons such as inappropriate probe placement, fewer than seven analyzable swallows, and sensor or thermal compensation malfunction.

Given the substantial clinical and diagnostic overlap between achalasia and GERD, misdiagnosis can result in inappropriate interventions that exacerbate symptoms. Here, we present the case of a patient with achalasia type 1 who was misdiagnosed with GERD and underwent an inappropriate Toupet fundoplication, who eventually required peroral endoscopic myotomy (POEM) as a salvage treatment to relieve their symptoms.

## Case presentation

A 69-year-old female with a past medical history of hiatal hernia, GERD (status: post-recent Toupet fundoplication), and hypothyroidism presented to the hospital with a two-week history of complete oral intolerance that began shortly after the fundoplication.

Approximately five months prior, she was evaluated by an outside gastroenterologist for a 10-year history of GERD, regurgitation, and solid food dysphagia. She was initiated on pantoprazole (40 mg daily) with minimal relief. One month later, EGD revealed diffuse esophageal dilation with white mucosa; no hiatal hernia was noted on retroflexion. Bougie dilation to 54 French (Fr) was performed, though no stricture, mass, or ulceration was reported. However, her dysphagia persisted after dilation.

**Figure 1:**
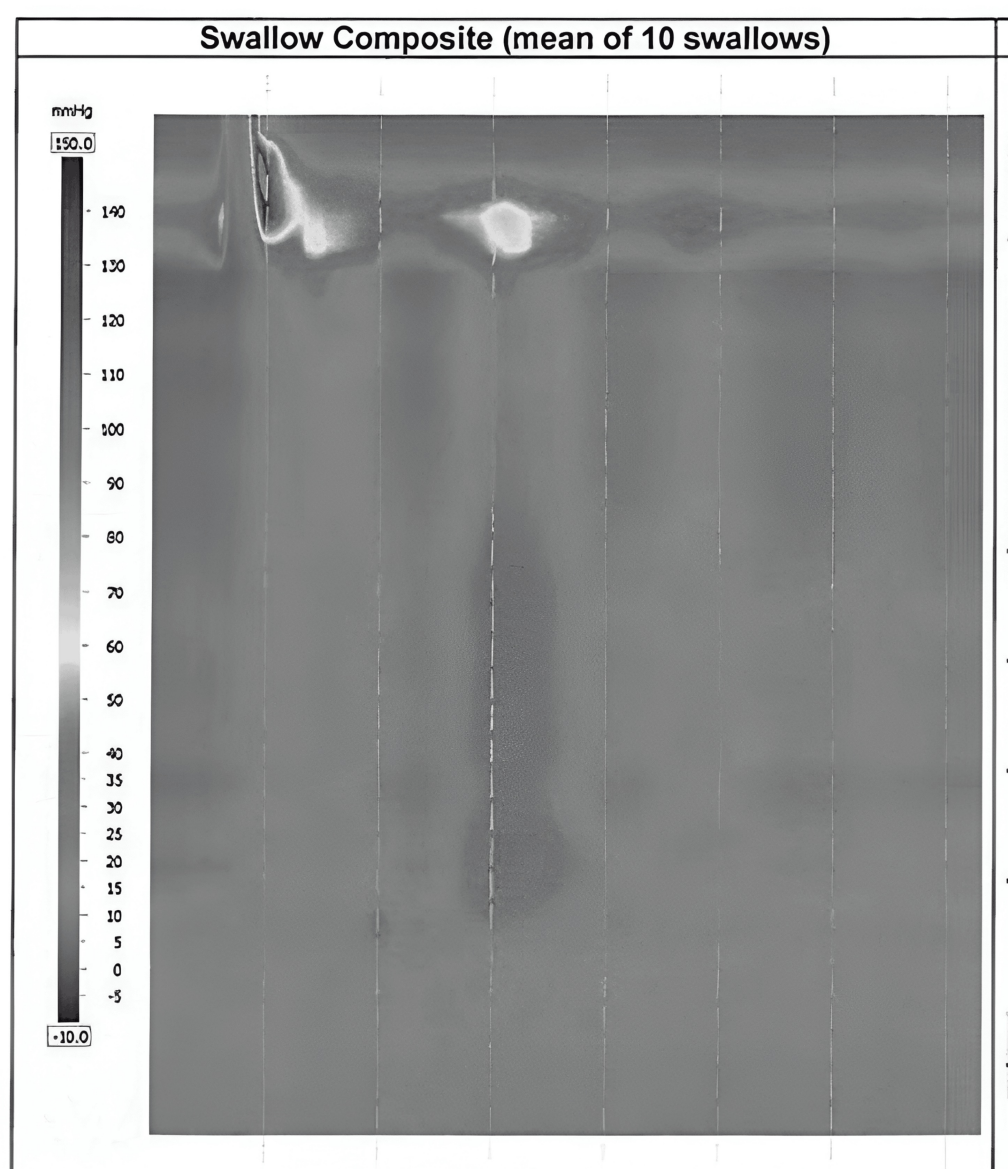
High-resolution manometry (10-swallow composite) showing ineffective esophageal motility with integrated relaxation pressure of 3.1.

One month later, HRM showed 100% ineffective swallows, with 50% failed swallows and an integrated relaxation pressure (IRP) of 3.1 mmHg (Figure 1). The patient was diagnosed with ineffective esophageal motility, corresponding with the Chicago classification of achalasia type 1. Achalasia was not considered at that time due to the normal range of LES relaxation on motility testing. She was continued on proton pump inhibitor (PPI) therapy, which provided some symptom relief, though nocturnal regurgitation persisted.

**Figure 2:**
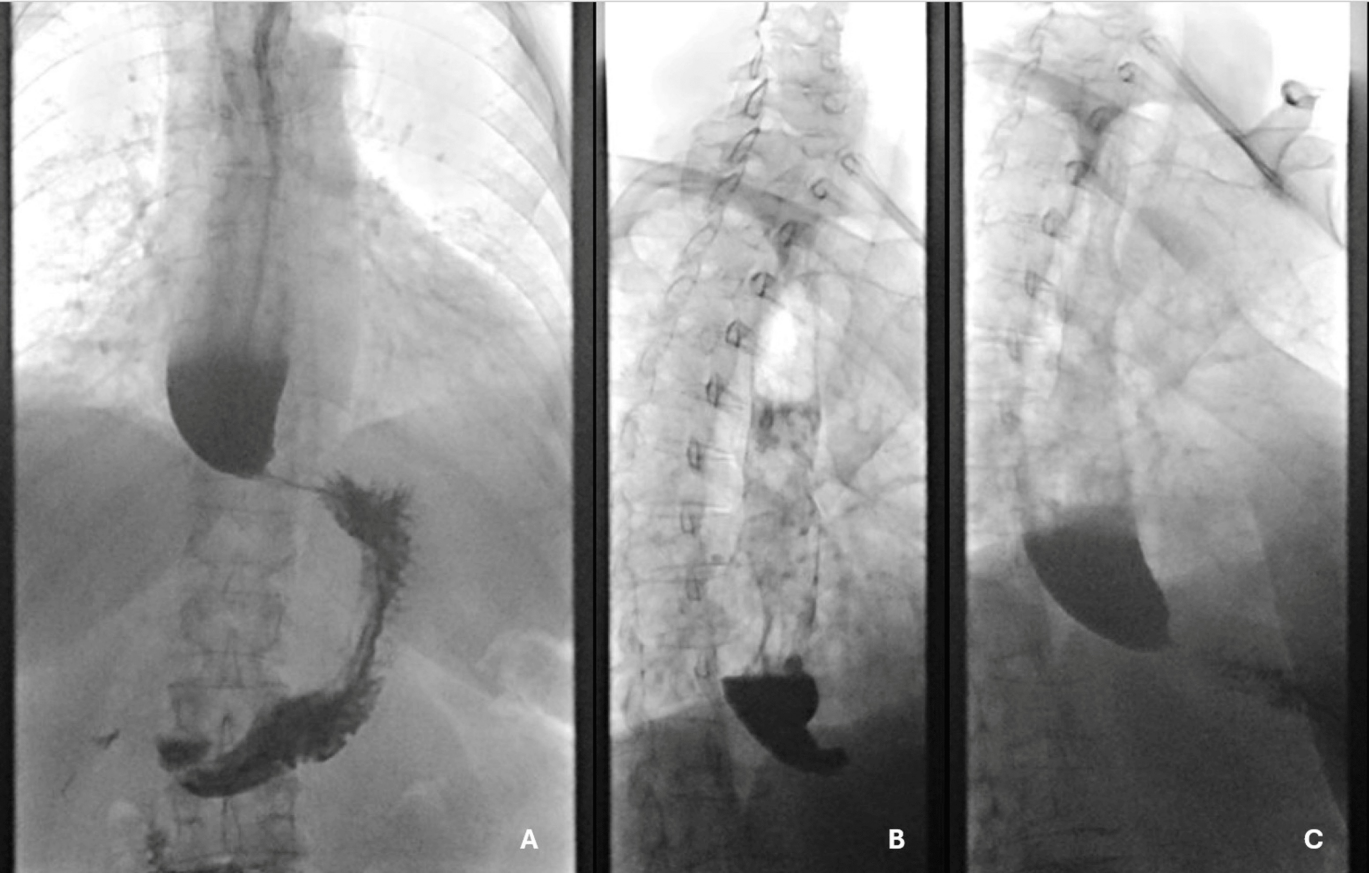
Timed barium esophagram showing a dilated esophagus tapering to a “bird’s beak”-type point, with a significant amount of retained contrast in the esophagus. Frame A is the beginning of study, B is the middle, and C is the end of the study.

Three months later, an upper gastrointestinal series (UGIS) revealed slow contrast emptying from a dilated esophagus tapering to a “bird’s beak”-type point, with significant retained contrast (Figure 2). A general surgeon at an outside facility reviewed the imaging and noted findings consistent with achalasia but found them inconsistent with the HRM results. Given the normal IRP, the working diagnosis remained reflux stricture, and the patient was scheduled for fundoplication with plans for potential future dilations once reflux was controlled.

Two months later, the patient underwent robotic hiatal hernia repair with a posterior 270-degree Toupet fundoplication. Intraoperatively, an inflamed distal esophagus with mucosal thickening and a significant transition point 2 to 3 cm above the Z-line was noted; the esophagus appeared normal below this area of inflammation. At her postoperative follow-up one week later, the patient reported worsening dysphagia with intolerance to her own saliva. She was admitted to the hospital for nasogastric (NG) tube placement and enteral feeding. The NG tube passed into the stomach without resistance, and she tolerated feeds but experienced several episodes of emesis. Given her persistent symptoms, she was transferred to our facility for evaluation.

At the time of transfer, her Eckardt score was 6 (dysphagia: 3, regurgitation: 2, chest pain: 1, weight loss: 0). A review of her prior endoscopic, motility, and radiographic findings raised concern for HRM catheter coiling and underlying achalasia, which was exacerbated further by the recent fundoplication. EGD revealed significant resistance at 44 cm during endoscope passage, requiring dilation and copious fluid suctioning. The esophageal and gastric mucosa were mildly inflamed and friable, and retroflexion in the stomach revealed evidence of the prior fundoplication. Endoluminal functional lumen imaging probe (EndoFLIP) showed a luminal diameter of 5.3 mm, distensibility of 0.7 mm^2^/mmHg, and a cross-sectional area (CSA) of 24 mm^2^ at the gastroesophageal junction (GEJ).

**Figure 3:**
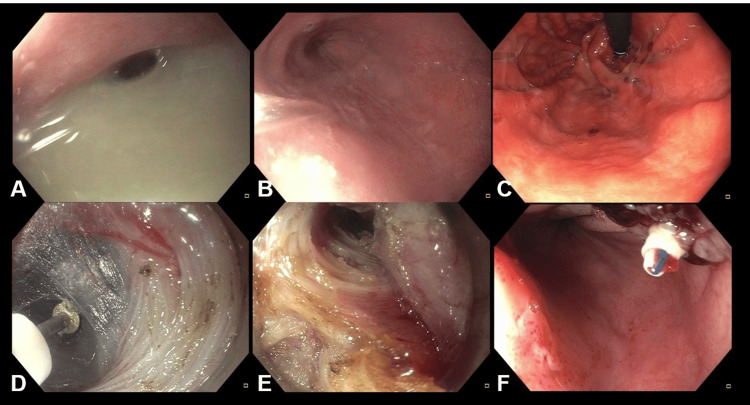
Endoscopic images of (A) copious fluid in the esophagus; (B) a dilated esophagus with puckered appearance of lower esophageal sphincter; (C) retroflexed view of stomach with evidence of prior fundoplication; (D) submucosal dissection prior to myotomy; (E) the myotomy site; (F) endoscopic suturing after myotomy.

The patient underwent POEM, with a posterior submucosal entry point made 10 cm proximal to the GEJ and dissection extending 3 cm past the GEJ. Circular muscle dissection began 1 cm distal to the mucosal entry, with 3 to 4 cm of the myotomy full thickness in the mid-tunnel (Figure 3). A post-POEM EndoFLIP confirmed treatment success, with an increased luminal diameter of 7.6 mm, distensibility of 2.4 mm^2^/mmHg, and CSA of 45 mm^2^.

**Figure 4:**
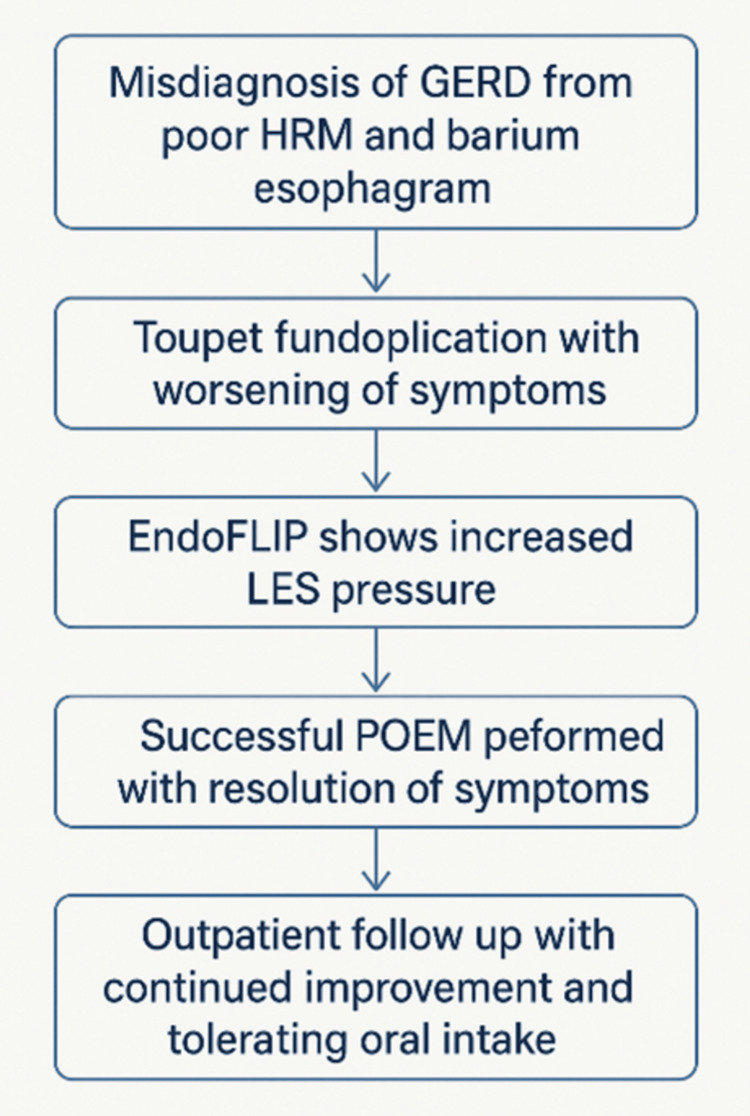
Summary timeline of events from misdiagnosis to resolution of symptoms. GERD: gastroesophageal reflux disease; HRM: high-resolution manometry; EndoFLIP: endoluminal functional lumen imaging probe; LES: lower esophageal sphincter; POEM: peroral endoscopic myotomy

On post-POEM day 1, a barium esophagram (BE) showed no evidence of a leak. The patient tolerated a clear liquid diet with minimal pain and no vomiting or regurgitation. Her diet was advanced to full liquids, which she tolerated well. She was discharged home on post-POEM day 5 on a full liquid diet and daily PPI therapy. At her 10-day follow-up visit, she was tolerating protein shakes without vomiting or regurgitation, with an Eckardt score improvement from 6 to 0. Five months after the procedure, she was tolerating a full diet and was transitioned back to her gastroenterologist for routine follow-up. A summary timeline of symptoms is shown in Figure 4.

## Discussion

Achalasia is an uncommon disease that is frequently misdiagnosed, as seen in our patient. Initial misdiagnosis occurs in nearly 50% of cases [4], often due to symptom variability and overlap with GERD. While simultaneous dysphagia to solids and liquids is a classic symptom of achalasia, it is only seen in approximately 75% of patients [5], with our patient experiencing dysphagia only to solids. Additionally, more than half of patients with achalasia report substernal chest pain and heartburn [6], symptoms commonly associated with GERD, while 37% of patients with erosive esophagitis present with dysphagia [7]. See Table 1 for comparisons between GERD and achalasia.

**Table 1:** Comparisons of typical symptoms and diagnostic evaluation between achalasia and GERD. GERD: gastroesophageal reflux disease; PPI: proton pump inhibitor; LES: lower esophageal sphincter

Category	GERD	Achalasia
Primary symptoms	Heartburn, acid regurgitation, chest discomfort	Dysphagia to liquids and solids, regurgitation of undigested food, chest pain
Symptom onset	Gradual - worsens with large meals, lying down, or spicy/fatty foods	Progressive over months to years - minimal changes to symptoms with food type or position
Relief with an antacid	Usually improves with antacids or PPI	No significant relief with acid suppression
Esophageal motility	Normal or hypotensive LES	Aperistalsis and impaired LES relaxation
Upper endoscopy findings	Esophagitis, strictures	Often, a normal or dilated esophagus with retained food or secretions
Barium swallow	May show reflux or hiatal hernia	"Bird-beak" tapering of the distal esophagus
Manometry findings	Normal or hypotensive peristalsis, low LES pressure	Absent peristalsis, incomplete LES relaxation
pH monitoring	Abnormal (increased acid exposure)	Normal (not acid-driven)
Definitive diagnosis	24-hour pH monitoring, response to PPI therapy	Esophageal manometry

EGD findings can also be misleading. A hallmark of achalasia is resistance to endoscope passage at the EGJ, but this may be misinterpreted as a peptic stricture in the setting of GERD. Furthermore, characteristic signs such as esophagus dilation with retained food may be absent in early achalasia. The recently developed CARS (Contents, Anatomy, Resistance, and Stasis) scoring system, which assesses contents, anatomy, resistance, and stasis of the esophagus, offers promise in improving EGD diagnostic capabilities, with preliminary studies showing high specificity for achalasia [8].

HRM remains the gold standard for diagnosing achalasia, though proper technique and probe placement are essential for accurate results and interpretation. The Chicago Classification 4.0 system standardizes HRM implementation and result interpretation, requiring proper probe calibration and placement to record esophageal motility [3]. According to the classification, achalasia is diagnosed when esophageal manometry shows increased median relaxation pressure (>15 mmHg) and 100% absent peristalsis [3]. However, even with a standardized technique and protocol, approximately 20% of HRM studies are imperfect [7]. Improper probe placement is a frequent issue, occurring in 2.0% to 13.2% of cases [4], particularly in patients with hiatal hernias or achalasia [7]. However, even with improper probe placement, HRM provides valuable information about esophageal peristalsis. In cases of incorrect positioning, blind observers still correctly diagnosed achalasia in 77% of cases, increasing to 94% when the clinical context was provided [7].

Several strategies can help mitigate HRM probe placement issues. For example, mild sedation can help with patient discomfort during probe placement with minimal effect on HRM results [9]. Ensuring adequate training of the practitioner placing the probe is also critical, with experts recommending that practitioners observe at least 20 studies and perform a minimum of 50 placements annually to maintain proficiency [10]. Additionally, direct visualization of probe placement using endoscopic assistance is the best way to improve probe positioning, particularly in patients with prior placement failures.

Even with ideal catheter placement, HRM may be flawed for other reasons, such as incomplete swallow protocols (i.e., fewer than seven evaluable swallows), sensor or thermal compensation malfunctions, or other miscellaneous artifacts [7]. Notably, achalasia is most frequently associated with incomplete swallow protocols, highlighting the irony that the very condition that HRM is designed to diagnose presents the greatest diagnostic challenges.

When HRM results are inconclusive or raise suspicion for an obstruction that does not fulfill achalasia criteria, the Chicago Classification 4.0 recommends a TBE or EndoFLIP to provide further diagnostic clarity [3]. TBE, a longstanding imaging modality for dysphagia, has high false-negative rates, especially when performed in community hospitals [5]. However, when performed at an esophageal center of excellence, TBE has a specificity of 92% [6]. To enhance diagnostic accuracy, a simple standardized TBE protocol was developed and is recommended to evaluate all patients with dysphagia [5]. In our case, a UGIS was performed instead of a TBE, but it provided strong evidence suggestive of achalasia. EndoFLIP, which measures pressure changes, diameter, and volume to provide a three-dimensional image of the esophageal lumen, is a high-sensitivity tool in diagnosing achalasia, with some studies reporting near 100% accuracy [11]. It is especially valuable in cases with equivocal HRM, as it is able to assess the motility of the LES by measuring the EGJ distensibility index and CSA. Furthermore, EndoFLIP is performed during EGD while a patient is sedated, making it a practical tool for patients unable to tolerate HRM. While the role of EndoFLIP in achalasia is still evolving, the American College of Gastroenterology guidelines suggest that it could be utilized in difficult cases before and after treatment [12]. In our case, EndoFLIP could have functioned as an arbiter before surgical referral, potentially preventing an unnecessary fundoplication.

This case highlights the need for the clinician to use multiple diagnostic modalities along with the clinical picture to help make the diagnosis, especially when there is discordance between the diagnostic studies. In this case, the contrast study was classic for achalasia findings, as were here endoscopic findings and clinical symptoms. It was only the IRP that did not fit the diagnosis of achalasia. Recognizing that HRM is not a perfect test, identifying its potential misinformation due to catheter misplacement can help avoid devastating errors in clinic management.

The other point highlighted by this case is the efficacy of POEM in treating post-fundoplication-related dysphagia. POEM showed promising results in cases where post-fundoplication dysphagia arises due to a secondary motility disorder, with 75% of patients regaining the ability to tolerate a normal diet without dysphagia or regurgitation [13]. Our case suggests that POEM can also be effective in patients who have undergone fundoplication due to unrecognized achalasia.

## Conclusions

Esophageal motility disorders can prove to be a diagnostic challenge despite increasing techniques. Achalasia and GERD share overlapping symptoms, and commonly used diagnostic modalities such as EGD and TBE may not accurately differentiate between them due to suboptimal sensitivity and specificity. HRM remains the gold standard for diagnosing achalasia, but technical challenges such as probe misplacement and esophageal pathology can hinder accuracy. In cases in which a patient’s diagnosis is still in question after HRM, additional testing with TBE or EndoFLIP may help elucidate a diagnosis. Notably, achalasia and GERD require fundamentally opposing treatments, and selecting one when the other is indicated can exacerbate symptoms and lead to adverse outcomes. For patients with post-fundoplication motility disorders, POEM may be a valuable therapeutic option to improve dysphagia and overall quality of life. Our case underscores the importance of reassessing a diagnosis prior to invasive treatment when workup yields conflicting data.
